# Association Between Sleep Microstructure and Incident Hypertension in a Population‐Based Sample: The HypnoLaus Study

**DOI:** 10.1161/JAHA.121.025828

**Published:** 2022-07-05

**Authors:** Mathieu Berger, Andrew Vakulin, Camila Hirotsu, Nicola Andrea Marchi, Geoffroy Solelhac, Virginie Bayon, Francesca Siclari, José Haba‐Rubio, Julien Vaucher, Peter Vollenweider, Pedro Marques‐Vidal, Bastien Lechat, Peter G. Catcheside, Danny J. Eckert, Robert J. Adams, Sarah Appleton, Raphael Heinzer

**Affiliations:** ^1^ Center for Investigation and Research in Sleep Department of Medicine Lausanne University Hospital and University of Lausanne Lausanne Switzerland; ^2^ Flinders Health and Medical Research Institute: Sleep Health/Adelaide Institute for Sleep Health Flinders University College of Medicine and Public Health Adelaide Adelaide SA Australia; ^3^ Department of Medicine Internal Medicine Lausanne University Hospital (CHUV) and University of Lausanne Lausanne Switzerland

**Keywords:** delta power, hypertension, power spectral density, sleep architecture, sleep structure, slow wave sleep, spindle, Epidemiology, Risk Factors, Hypertension

## Abstract

**Background:**

Poor sleep quality is associated with increased incident hypertension. However, few studies have investigated the impact of objective sleep structure parameters on hypertension. This study investigated the association between sleep macrostructural and microstructural parameters and incident hypertension in a middle‐ to older‐aged sample.

**Methods and Results:**

Participants from the HypnoLaus population‐based cohort without hypertension at baseline were included. Participants had at‐home polysomnography at baseline, allowing assessment of sleep macrostructure (nonrapid eye movement sleep stages 1, 2, and 3; rapid eye movement sleep stages; and total sleep time) and microstructure including power spectral density of electroencephalogram in nonrapid eye movement sleep and spindles characteristics (density, duration, frequency, amplitude) in nonrapid eye movement sleep stage 2. Associations between sleep macrostructure and microstructure parameters at baseline and incident clinical hypertension over a mean follow‐up of 5.2 years were assessed with multiple‐adjusted logistic regression. A total of 1172 participants (42% men; age 55±10 years) were included. Of these, 198 (17%) developed hypertension. After adjustment for confounders, no sleep macrostructure features were associated with incident hypertension. However, low absolute delta and sigma power were significantly associated with incident hypertension where participants in the lowest quartile of delta and sigma had a 1.69‐fold (95% CI, 1.00–2.89) and 1.72‐fold (95% CI, 1.05–2.82) increased risk of incident hypertension, respectively, versus those in the highest quartile. Lower spindle density (odds ratio, 0.87; 95% CI, 0.76–0.99) and amplitude (odds ratio, 0.98; 95% CI, 0.95–1.00) were also associated with higher incident hypertension.

**Conclusions:**

Sleep microstructure is associated with incident hypertension. Slow‐wave activity and sleep spindles, 2 hallmarks of objective sleep continuity and quality, were inversely and consistently associated with incident hypertension. This supports the protective role of sleep continuity in the development of hypertension.

**Nonstandard Abbreviations and Acronyms**
AASM: American Academy of Sleep Medicine
N1: nonrapid eye movement sleep stage 1
N2: nonrapid; eye movement sleep stage 2
N3: nonrapid eye movement sleep stage 3
NREM: nonrapid eye movement
PSD: power spectral density
SWAN: Study of Women’s Health Across the Nation
SWS: slow wave sleep

**Clinical Perspective**

**What Is New?**

- In contrast to previous data on the relationship between sleep structure and hypertension, this study performed a more in‐depth analysis, including comprehensive objective sleep microstructural and more conventional sleep macrostructural parameters.
- We found that slow wave activity and sleep spindles, 2 hallmarks of sleep continuity and objective sleep quality, were inversely associated with incident hypertension and were robust to multiple adjustments for potential confounders, while sleep macrostructure was not associated with the development of hypertension.

**What Are the Clinical Implications?**

- These findings reinforce the concept of a protective role of sleep continuity in the development of hypertension, implying that general practitioners and cardiologists should consider sleep quality when conducting clinical hypertension risk assessment.

High blood pressure (BP) is a leading risk factor for cardiovascular disease–related mortality.^1^ Sympathetic nervous system activity and BP tend to decrease with the progressive deepening of nonrapid eye movement (NREM) sleep^2^ and return to levels similar to wakefulness during rapid eye movement sleep.^3^ BP also demonstrates transient changes over the course of sleep, such as a gradual rise during airway obstruction events and surges following arousal and awakening events.^4^ Several epidemiological studies have demonstrated that reduced BP dipping or “nondipping” during sleep increases cardiovascular risk.^5^, ^6^, ^7^ In addition, growing evidence suggests that short sleep duration and subjective poor sleep quality are associated with cardiovascular comorbidities.^8^, ^9^, ^10^, ^11^, ^12^ Experimental studies suggest that the magnitude of nocturnal BP dipping is significantly altered by deep slow wave sleep (SWS; or NREM sleep stage 3 [N3]) deprivation in young healthy individuals with normotension.^13^ The importance of SWS in the modulation of BP was further supported in 2 community‐based cohorts showing that the percentage of SWS was inversely associated with incident hypertension.^14^, ^15^

However, conventional “macroscopic” sleep stage scoring represents a superficial simplification of electroencephalogram (EEG) temporospatial and frequency domains, and may vary between scorers because of visual‐based rules that remain subjective.^16^ Thus, quantification of sleep‐related brain oscillations via power spectral density (PSD) analysis of EEG signals have been developed, allowing for more objective and finer‐grained analysis of sleep microstructure. This method allows the decomposition of EEG brain waves across a range of power frequency bands from slow wave activity (delta EEG power, 1–4 Hz) to fast frequency activity (beta EEG power, 18–30 Hz) using fast Fourier transform algorithms. SWS is characterized at the microscopic level by the presence of high delta power, which reflects the presence of high‐amplitude slow waves typical of deep sleep. Several studies suggest that quantitative EEG may provide more sensitive markers for identifying patient phenotypes at risk for adverse health outcomes compared with conventional sleep scoring.^17^, ^18^, ^19^, ^20^ However, few studies have investigated the association between sleep microstructure and future development of hypertension. One study performed in a small sample of middle‐aged women showed that those with low delta power during NREM sleep may be at increased risk of developing hypertension.^21^ However, obstructive sleep apnea was not controlled for, which clearly impacts quantitative EEG,^19^ and thus may have confounded the association with hypertension.

Furthermore, novel computed algorithms allow more in‐depth analysis of EEG graphical elements, such as spindle characteristics during NREM sleep stage 2 (N2). Although previous studies have shown that lower spindle density could determine neural dysfunction and cognitive decline,^22^, ^23^ none have investigated the impact of spindle characteristics in the development of hypertension.

Therefore, the aim of this study was to investigate the potential association between both sleep macrostructure and microstructure and incident hypertension in middle‐ to older‐aged adults of the general population.

## Methods

### Data Availability

Because of the sensitivity of the data and the lack of consent for online posting, individual data cannot be made accessible. Only metadata will be made available in digital repositories on reasonable request. Metadata requests can also be made via the study website: https://www.colaus‐psycolaus.ch.

### Study Design and Population

The sample was derived from the prospective CoLaus|PsyCoLaus population‐based cohort^24^, ^25^ and HypnoLaus, a nested study, designed to assess the prevalence and determinants of sleep disorders in participants randomly selected from the city of Lausanne, Switzerland. The design, sampling, and procedures of the study have been described elsewhere.^26^ Briefly, 2162 individuals from CoLaus|PsyCoLaus underwent a home‐based polysomnography and multiple clinical assessments, including demographic, medical history, anthropometric, and office BP measurements, between 2009 and 2012 (considered as baseline hereafter). Between 2014 and 2017, participants underwent a second examination in which clinical parameters were reassessed. Inclusion criteria were: (1) absence of hypertension at baseline, (2) complete data for hypertension diagnosis at baseline and follow‐up, and (3) full polysomnography performed at baseline. HypnoLaus and CoLaus|PsyCoLaus were approved by the ethics committee of the Vaud Canton (CER‐VD n° PB_2018‐00038 (239/09)) and all participants provided written informed consent.

### BP Measurements

BP was assessed in triplicate on the left arm at 5‐minute intervals with the participant seated and resting for at least 10 minutes using calibrated automated oscillometric sphygmomanometers (Omron HEM‐907). The mean of the second and third measurements was used for analysis, as recommended.^27^ Hypertension was defined as systolic BP ≥140 mm Hg and/or diastolic BP ≥90 mm Hg and/or antihypertensive medication use.

### Outcome

Incident hypertension was defined as the absence of hypertension at the time of polysomnography (baseline) and the development of hypertension assessed at the clinical follow‐up of the CoLaus|PsyCoLaus cohort in 2014–2017.

### Clinical Assessment and Covariates

Information on sociodemographic characteristics and medical and treatment history was obtained by trained interviewers using standardized questionnaires at baseline. Alcohol consumption was defined using weekly mean consumption of standard drinks containing 10 g of alcohol. Food sodium content was assessed using a validated food frequency questionnaire querying the consumption of 97 different food items including portion size over the previous 4 weeks. Medication was coded according to World Health Organization Anatomical, Therapeutic, Chemical classification. Psychotropic drugs included antidepressants, anxiolytics, antipsychotics, benzodiazepines, and “Z‐drugs.” Body weight and height were measured with participants standing without shoes in light clothes to calculate body mass index. Diabetes was defined as fasting blood glucose ≥7 mmol/L and/or use of antidiabetic drugs.^28^ Reumathoid arthritis was self‐reported. Restless leg syndrome was investigated using the International Restless Legs Syndrome Study Group criteria.^29^

Self‐reported sleep quality was assessed with the Pittsburgh Sleep Quality Index,^30^ where a score >5 indicates poor sleep quality. Sleepiness was evaluated with the Epworth Sleepiness Scale,^31^ where a score >10 indicates excessive daytime sleepiness.

### Polysomnography

Home‐based polysomnography (Titanium) was conducted with multiple EEG leads (C3, C4, F3, F4, O1, and O2; 256 Hz sampling rate). Polysomnography setup specifications followed the 2007 American Academy Sleep Medicine (AASM) recommendations.^32^ Polysomnography data included EEG (central, occipital, frontal), electrooculogram (right and left eyes), chin and anterior tibialis electromyogram, ECG, nasal pressure, thoracic and abdominal effort bands, oximetry, snoring, and body position signals. Manual scoring of sleep stage was performed using the 2007 AASM criteria,^32^ while respiratory events were scored according to the 2012 AASM criteria.^33^ Obstructive sleep apnea was defined by an apnea‐hypopnea index ≥15 events per hour.

### EEG PSD Analysis

Detailed descriptions of EEG PSD data preprocessing including artifact detection and management are described elsewhere.^34^ Synchronized European data format and sleep stage files were generated using Embla RemLogic Polysomnography Software (Natus Medical, Incorporated). PSD values were calculated in C3‐M2 using fast Fourier transformation and the Welch algorithm on artifact‐free consecutive, nonoverlapping 6‐second epochs (Hamming windows, 8 segments, 50% overlap) and used to compute absolute signal power (μV^2^) in typical frequency bands including delta (1–4 Hz), theta (5–8 Hz), alpha (8–12 Hz), sigma (12–16 Hz), and beta (18–30 Hz).

### Spindle Characteristics

**Figure 1:**
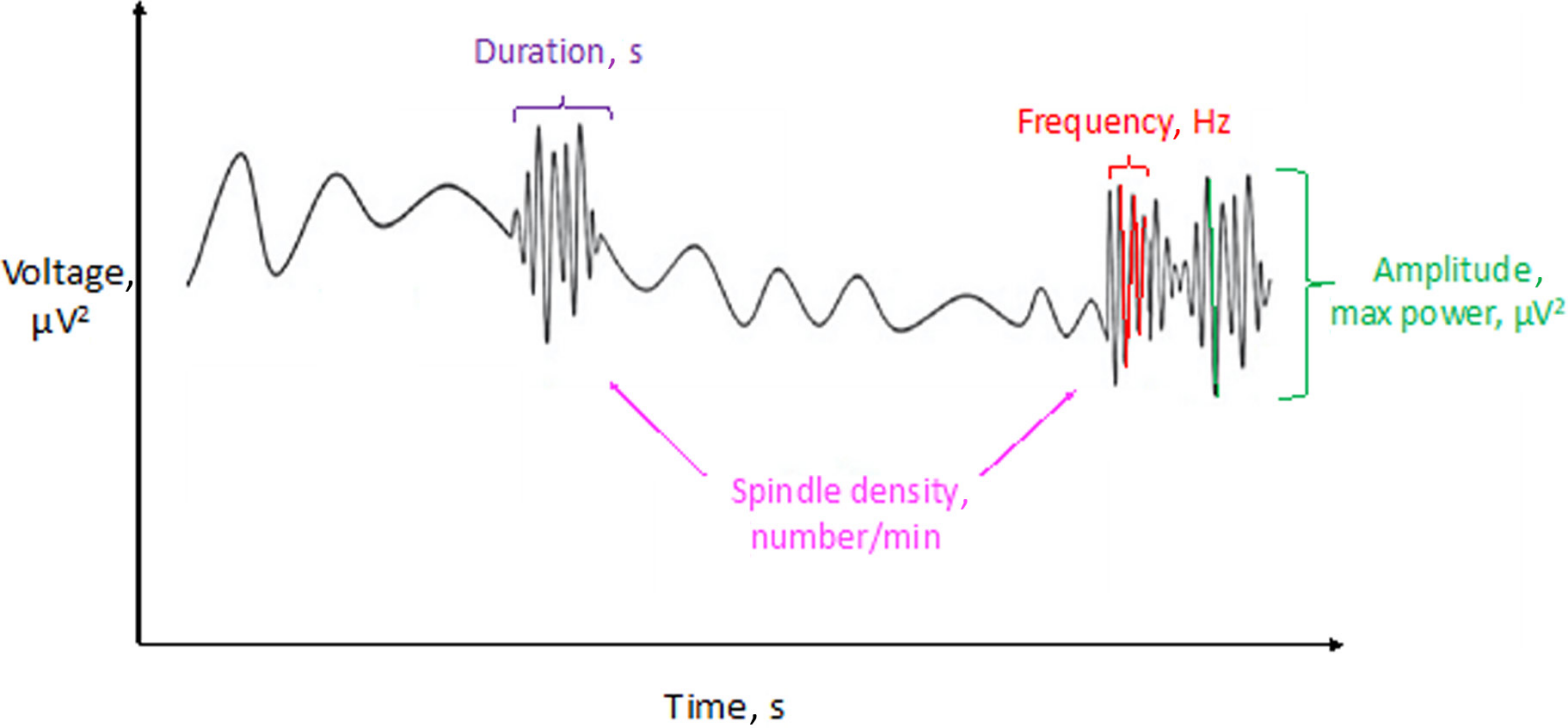
Sleep spindle characteristics.

Spindles were separately identified in the 10‐Hz to 16‐Hz range in central derivations (C3 and C4) during N2 based on an automatic algorithm, as previously described.^35^ The following spindle characteristics were computed for each detected spindle: spindle density defined as total number of spindles in N2 divided by the N2 time in minutes; spindle frequency, defined as the frequency with the highest power in the 10‐Hz to 16‐Hz range; spindle amplitude, defined as the highest power within the spindle (expressed in microvolts squared; µV^2^); spindle duration, defined as the average spindle length (second) of each spindle event; and percentage of fast spindles (12‐ to 16‐Hz range) of all identified spindles (Figure 1). Spindle characteristics were then averaged across both C3‐M2 and C4‐M1 when artifact‐free.

### Statistical Analysis

Descriptive and inferential statistics were performed using IBM SPSS Statistics version 26. Data are presented as number (percentage) of patients, median (interquartile range [IQR]), or mean±SD, unless otherwise stated. Data distribution was graphically assessed through normal Q‐Q plot. Pearson chi‐square test, independent samples *t* test or Mann‐Whitney pairwise comparisons were used as appropriate to compare differences in baseline characteristics based on the presence or absence of hypertension. Associations between sleep macrostructure and microstructure and the incidence of hypertension were evaluated using unadjusted (model 0) and multivariable‐adjusted logistic regression (model 1 and 2).

Locally weighted scatterplot smoothing was drawn to check the assumption of linearity for the logit of each continuous independent variable. In the case of nonlinearity, continuous variables were transformed into categorical variables based on clinically relevant cutoffs or median. Model 1 was adjusted for age (continuous), sex (categorical), and body mass index (continuous), while model 2 was additionally adjusted for alcohol consumption (continuous), baseline systolic BP (continuous), diabetes (categorical), dyslipidemia (categorical), obstructive sleep apnea (categorical, ie, apnea‐hypopnea index >15 events per hour), sleep efficiency (continuous), and duration of follow‐up (continuous). These confounding factors were included in the multivariate models either because of significant difference in bivariate analysis or because of their recognized clinical associations with hypertension (sex, alcohol). Since patients who developed hypertension had higher BP at baseline, baseline systolic BP was included as a covariate in model 2. Results are presented as odds ratio (ORs) and 95% CIs. When a sleep macrostructure or microstructure continuous parameter was significantly associated with incident hypertension in the fully adjusted model, it was further categorized into quartiles to account for potential nonlinear associations.

To facilitate interpretation, significant continuous scale results in fully adjusted models were further categorized into quartiles. As the association between spindle density and incident hypertension appeared nonlinear in quartile analysis, a complementary restricted cubic spline regression was performed using the rms package in R. In secondary analysis, given the a priori hypothesis that sleep quality may vary according to sex,^36^ we tested the interaction between sex and SWS, sleep microstructure, and spindles characteristics. Statistical significance was defined as a 2‐sided *P* value of <0.05.

## Results

**Figure 2:**
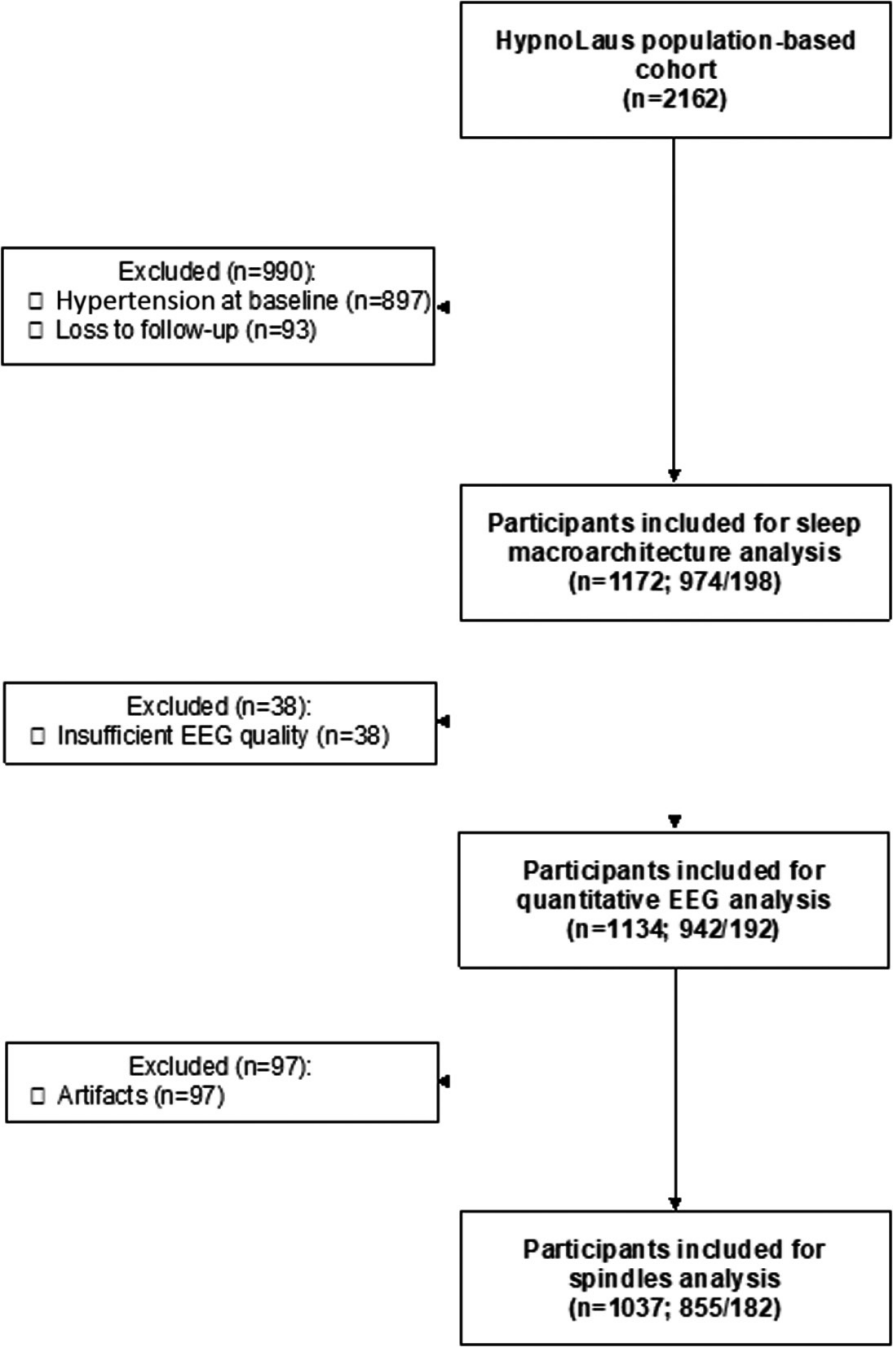
Study flow chart. n/n indicates number of participants without hypertension at follow‐up/number of participants who developed hypertension during follow‐up. EEG indicates electroencephalogram.

Of the 2162 participants included in HypnoLaus, 1172 who had hypertension at baseline with complete polysomnography and known hypertension status at follow‐up were included in this analysis (Figure 2). Approximatively 17% (n=198) of the sample developed hypertension during a mean±SD follow‐up of 5.2±0.6 years (minimum: 2.2 years; maximum: 7.8 years). Participants who developed hypertension were older (median age, 56 years [IQR, 49–66 years] versus 52 years [47–62 years]), with higher body mass index (median body mass index, 26.2 kg.m^−2^ [IQR, 23.6–28.8 kg.m^−2^] versus 24.5 kg.m^−2^ [IQR, 22.2–26.9 kg.m^−2^]) and more comorbidities than those who did not (Table 1). Regarding objective sleep, although participants who developed hypertension during follow‐up had similar TST to those without hypertension, there was a clinically nonrelevant but statistically significant difference in sleep efficiency (median sleep efficiency, 89.0% [IQR, 80.1%–93.0%] versus 89.9% [IQR, 84.2%–93.5%]; *P*=0.024). Moreover, patients who developed hypertension had higher arousal index, apnea‐hypopnea index, and hypoxemic parameters compared with those without hypertension (Table 1). Last, patients who developed hypertension had higher BP at baseline compared with those without hypertension (mean±SD systolic BP, 126±9 mm Hg versus 116±11 mm Hg; *P*<0.001).

**Table 1:** Baseline Demographic and Clinical Characteristics in Patients With and Without Incident Hypertension

	No hypertension	Incident hypertension	*P* value	No.
	(n=974)	(n=198)		
Age, y	52 (47–62)	56 (49–66)	0.001*	1172
Women, %	549 (58.5%)	105 (54.7%)	0.334	1172
BMI, kg/m^2^	24.5 (22.2–26.9)	26.2 (23.6–28.8)	<0.001*	1166
SBP, mm Hg	116±11	126±9	<0.001*	1172
DBP, mm Hg	73±8	79±7	<0.001*	1172
Alcohol consumption^†^	4 (1–8)	4 (1–10)	0.323	1172
Food sodium content			0.587	1111
Medication use, %				
Antidiabetic	8 (0.9%)	7 (3.6%)	0.002*	1172
Lipid‐lowering	57 (6.1%)	31 (16.1%)	<0.001*	1172
Psychotropic^‡^	13.1 (128)	13.1 (26)	0.997	1172
Comorbidities, %				
Diabetes	29 (3.1%)	18 (9.4%)	<0.001*	1171
Dyslipidemia	191 (20.4%)	61 (31.8%)	0.001*	1171
OSA^§^	230 (24.5%)	70 (36.5%)	0.001*	1172
RLS	148 (15.2%)	26 (13.1%)	0.457	1172
Arthritis	21 (2.2%)	3 (1.5%)	0.784	1172
Smoking, %				1159
Current smoker	190 (20.5%)	39 (20.6%)	0.863	
Ex‐smoker	332 (35.7%)	71 (37.6%)		
Excessive daytime sleepiness^||^	124 (13.7%)	24 (13.0%)	0.780	1087
Subjective sleep				
PSQI score	5.0±3.2	4.9±3.2	0.809	1018
Poor sleep quality^¶^	299 (34.9%)	57 (35.2%)	0.950	1018
Objective sleep				
TST, min	408±70	408±73	0.970	1172
Sleep efficiency, %	89.9 (84.2–93.5)	89.0 (80.1–93.0)	0.024*	1172
Arousal index, events per h	17.3 (12.7–23.7)	19.7 (14.3–26.9)	0.002*	1172
AHI, events per h	7.1 (2.8–14.8)	9.9 (3.8–21.0)	<0.001*	1172
ODI, events per h	6.7 (3.0–14.0)	10.3 (4.1–19.2)	<0.001*	1172
T90, %	0 (0–0.5)	0.1 (0–1.4)	<0.001*	1163
Mean SpO_2_, %	94.8 (93.7–95.7)	94.4 (93.3–95.4)	0.003*	1171
PLMSI, events per h	1.0 (0–11.6)	1.5 (0–15.7)	0.320	1172

Data are presented as number (percentage), median (interquartile range), or mean±SD, unless otherwise stated. AHI indicates apnea‐hypopnea index; BMI, body mass index; DBP, diastolic blood pressure; ODI, oxygen desaturation index; PLMSI, periodic leg movements during sleep; RLS, restless leg syndrome; SBP, systolic blood pressure; SpO_2_, oxygen saturation; TST, total sleep time; and T90, percentage of total sleep time with oxygen saturation <90%.

* Indicates significant *P*‐values.

^†^ Alcohol consumption was defined as weekly mean consumption of standard drinks containing 10 g of alcohol.

^‡^ Psychotropic drugs included antidepressants, hypnotics, anxiolytics, antipsychotics, benzodiazepines, and “Z‐drugs.”

^§^ Obstructive sleep apnea (OSA) was defined by an apnea‐hypopnea index ≥15 per hour.

^||^ Excessive daytime sleepiness was defined by an Epworth Sleepiness Scale score ≥11.

^¶^ Poor sleep quality was defined by a Pittsburgh Sleep Quality Index (PSQI) score ≥6.

### Association of Sleep Macrostructure With Incident Hypertension

A higher percentage of NREM sleep stage 1 (N1) sleep (mean difference, +1.4; 95% CI, 0.4–2.3 [*P*=0.002]) and a lower percentage of N3 sleep (mean difference, −1.8; 95% CI, −3.0–−0.5 [*P*=0.008]) in participants who developed hypertension was identified in bivariate analysis (Table 2). After multiple adjustments, no significant associations were found between sleep macrostructure and incident hypertension (Table 3).

**Table 2:** EEG Power Spectral Density During NREM Sleep (N1, N2, N3) and N2 Spindle Characteristics in Patients With and Without Incident Hypertension

	No hypertension	Incident hypertension	*P* value	No.
	(n=939)	(n=192)		
Sleep macrostructure				
N1 sleep, %	9.2 (7.0–13.0)	11.0 (7.2–15.6)	0.002*	1172
N2 sleep, %	45.8±9.8	45.9±9.4	0.870	1172
N3 sleep, %	21.0±8.2	19.3±7.4	0.008*	1172
REM sleep, %	22.6±5.7	22.8±5.4	0.583	1172
Sleep microstructure				
Delta, µV^2^	92.3 (66.3–123.8)	80.7 (60.5–107.9)	0.002*	1134
Theta, µV^2^	12.5 (9.1–17.5)	11.5 (8.2–16.0)	0.043*	1134
Alpha, µV^2^	10.5 (7.2–15.3)	9.0 (6.4–14.1)	0.003*	1134
Sigma, µV^2^	5.4 (3.8–7.7)	4.8 (3.1–7.1)	0.001*	1134
Beta, µV^2^	1.9 (1.4–2.6)	1.7 (1.3–2.4)	0.083	1134
EEG activation index (log (beta/delta))	−1.66±0.27	−1.64±0.23	0.279	1134
N2 spindle characteristics^†^				
Density, min^−1^	2.8±1.4	2.4±1.5	0.002*	1037
Amplitude, µV^2^	28.1±8.1	26.2±8.2	0.004*	1037
Frequency, Hz	12.4±0.5	12.4±0.6	0.211	1037
Duration, s	1.33±0.06	1.33±0.07	0.642	1037
Fast spindles, %	63.7±18.3	61.0±20.2	0.095	1037

EEG indicates electroencephalogram; N1, nonrapid eye movment sleep stage 1; N2, nonrapid eye movment sleep stage 2; N3, nonrapid eye movment sleep stage 3; NREM, nonrapid eye movement; and REM, rapid eye movement.

* Indicates significant *P*‐values.

^†^ A total of 1037 of the 1131 participants had complete data for spindle analysis.

**Table 3:** Incident Hypertension According to Subjective and Objective Sleep Parameters

	Crude		Model 1		Model 2	
	OR (95% CI)	*P* value	OR (95% CI)	*P* value	OR (95% CI)	*P* value
Subjective sleep quality (each 1‐PSQI point increase)	0.99 (0.94–1.05)	0.809	0.99 (0.94–1.05)	0.840	1.00 (0.95–1.06)	0.914
Sleep macrostructure						
N1 (each 1% increase)	1.03 (1.01–1.06)	0.005*	1.02 (0.99–1.04)	0.174	1.01 (0.98–1.04)	0.678
N2 (each 1% increase)	1.00 (0.99–1.02)	0.738	0.99 (0.98–1.01)	0.496	1.00 (0.98–1.01)	0.716
N3 (each 1% increase)	0.97 (0.95–0.99)	0.007*	0.99 (0.97–1.01)	0.180	0.99 (0.97–1.01)	0.262
REM (each 1% increase)	1.00 (0.98–1.03)	0.904	1.02 (0.99–1.05)	0.130	1.03 (1.00–1.06)	0.083
Sleep microstructure (absolute PSD)						
Delta power (each 10‐µV^2^ increase)	0.95 (0.91–0.98)	0.003*	0.96 (0.93–1.00)	0.043*	0.96 (0.92–1.00)	0.040*
Theta power (each 1‐µV^2^ increase)	0.99 (0.97–1.01)	0.166	0.99 (0.97–1.01)	0.232	0.99 (0.97–1.01)	0.325
Alpha power (each 1‐µV^2^ increase)	0.97 (0.95–0.99)	0.026*	0.98 (0.96–1.00)	0.055	0.98 (0.96–1.01)	0.113
Sigma power (each 1‐µV^2^ increase)	0.93 (0.88–0.98)	0.005*	0.94 (0.89–0.99)	0.022*	0.94 (0.89–0.99)	0.018*
Beta power (each 1‐µV^2^ increase)	0.96 (0.89–1.04)	0.298	0.96 (0.88–1.04)	0.309	0.95 (0.87–1.04)	0.256
EEG activation index	1.37 (0.77–2.44)	0.279	0.87 (0.52–1.82)	0.932	0.98 (0.50–1.96)	0.975
N2 spindle characteristics						
Density (each 1‐spindle.min^−1^ increase)	0.83 (0.74–0.94)	0.002*	0.87 (0.77–0.99)	0.027*	0.87 (0.76–0.99)	0.032*
Amplitude (each 1‐µV^2^ increase)	0.97 (0.95–0.99)	0.004*	0.98 (0.96–1.00)	0.041*	0.98 (0.95–1.00)	0.049*
Frequency (each 1‐Hz increase)	0.83 (0.62–1.11)	0.211	0.95 (0.70–1.29)	0.734	0.88 (0.64–1.21)	0.427
Duration (each 1‐s increase)	1.97 (0.16–23.9)	0.596	1.62 (0.13–19.8)	0.705	2.98 (0.23–38.6)	0.403
Fast spindles (each 1% increase), %	0.99 (0.98–1.00)	0.075	1.00 (0.99–1.01)	0.371	1.00 (0.99–1.01)	0.275

Data were analyzed using multivariable‐adjusted logistic regression. EEG indicates electroencephalogram; NREM, nonrapid eye movement; N1, nonrapid eye movement sleep stage 1; N2, nonrapid eye movement sleep stage 2; N3, nonrapid eye movement sleep stage 3; OR, odds ratio; PSD, power spectral density; PSQI, Pittsburg Sleep Quality Index; and REM, rapid eye movement.

Model 1 was adjusted for age, sex, and body mass index; and model 2 was additionally adjusted for alcohol consumption, systolic blood pressure, diabetes, dyslipidemia, obstructive sleep apnea, sleep efficiency, and duration of follow‐up.

* Indicates significant *P*‐values.

### Associations of EEG PSD With Incident Hypertension

**Figure 3:**
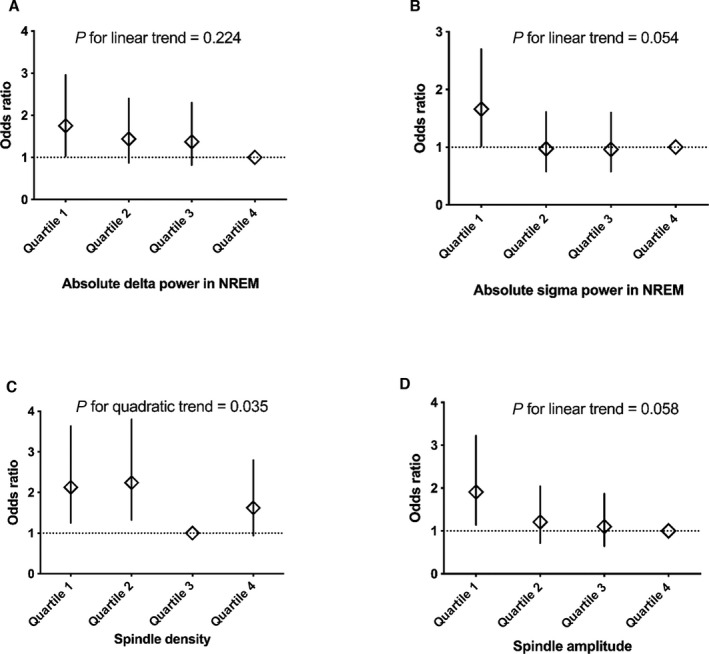
Association between incident hypertension at 5 years of follow‐up and: (**A**) quartiles of delta power in nonrapid eye movement sleep (NREM); (**B**) quartiles of sigma power in NREM; (**C**) quartiles of spindle density in NREM sleep stage 2 (N2); and (**D**) quartiles of spindle amplitude in N2. Results were analyzed using multivariable logistic regression with adjustment for baseline age, body mass index, sex, alcohol consumption, systolic blood pressure, diabetes, dyslipidemia, obstructive sleep apnea, sleep efficiency, and duration of follow‐up (model 2).

Bivariate analysis showed that participants who developed hypertension had lower EEG power in almost all power frequency bands during NREM sleep, except in the beta band (Table 2). After adjustment for age, sex, and body mass index (model 1), only the absolute delta power (OR, 0.96; 95% CI, 0.93–1.00) and absolute sigma power (OR, 0.94; 95% CI, 0.89–0.99) remained associated with incident hypertension (Table 3). These associations persisted in the fully adjusted model (model 2; Table 3). When delta and sigma were categorized in quartiles, participants with delta and sigma values in the lowest quartile (quartile 1) had an ≈1.7‐fold increased risk of developing hypertension during follow‐up compared with those who had delta and sigma values in the highest quartile (quartile 4) (OR, 1.69 [95% CI, 1.00–2.89] for quartile 1 delta and 1.72 [95% CI, 1.05–2.82] for quartile 1 sigma compared with quartile 4 delta and sigma, respectively) (Figure 3).

### Associations of Spindle Characteristics and Incident Hypertension

Participants who developed hypertension had lower spindle density (mean difference, −0.37; 95% CI, −0.61 to −0.14 min^−1^ [*P*=0.002]) and lower spindle amplitude (mean difference, −1.92; 95% CI, −3.22 to −0.61 µV^2^) in bivariate analysis compared with those who did not develop hypertension (Table 2). After multiple adjustments (models 1 and 2), both spindle density (OR, 0.87; 95% CI, 0.76–0.99) and spindle amplitude (OR, 0.98; 95% CI, 0.95–1.00) remained significantly associated with incident hypertension (Table 3). Quartile analysis suggested a possible U‐shaped relationship between spindle density and incident hypertension (*P* for quadratic trend=0.039). Participants with spindle density in quartile 1 and quartile 2 had a 2.13‐fold (95% CI, 1.25–3.63) and 2.24‐fold (95% CI, 1.32–3.80) increased risk of developing hypertension, respectively, compared with quartile 3, while the fourth quartile did not differ significantly (OR, 1.62; 95% CI, 0.94–2.80) (Figure 3). However, the U‐shaped association did not remain when spindle density was modeled as a continuous variable using restricted cubic spline (*P*=0.085, Figure S1). In addition, categorization of spindle amplitude in quartiles produced a linear relationship, with participants in the first quartile having a significant increased risk of incident hypertension compared with participants in the fourth quartile (OR, 1.91; 95% CI, 1.14–3.22 [*P* for trend=0.058]) (Figure 3).

### Secondary Analysis

No interaction of either sex, age, OSA, or excessive daytime sleepiness was found in the relationship between incident hypertension and SWS, delta in NREM, sigma in NREM, spindle density, and spindle amplitude (Table 4).

**Table 4:** Interaction Analysis Between Sex, Age, OSA, Excessive Daytime Sleepiness, and Sleep Parameters for Incident Hypertension

	Sex interaction		Age interaction		OSA interaction		EDS interaction	
	OR (95% CI)	*P* value	OR (95% CI)	*P* value	OR (95% CI)	*P* value	OR (95% CI)	*P* value
SWS	1.01 (0.97–1.06)	0.642	1.03 (0.98–1.08)	0.208	1.04 (0.99–1.08)	0.133	1.02 (0.95–1.09)	0.649
Delta in NREM	1.00 (0.99–1.01)	0.538	1.00 (0.99–1.01)	0.152	1.01 (1.00–1.01)	0.166	1.00 (0.99–1.01)	0.956
Sigma in NREM	0.97 (0.86–1.09)	0.599	1.01 (0.90–1.12)	0.891	1.00 (0.89–1.12)	0.997	0.99 (0.83–1.20)	0.944
Spindle density	0.86 (0.65–1.12)	0.248	0.94 (0.73–1.22)	0.661	0.80 (0.59–1.08)	0.142	1.06 (0.72–1.56)	0.762
Spindle amplitude	0.97 (0.92–1.02)	0.225	1.01 (0.96–1.06)	0.702	1.01 (0.95–1.06)	0.860	0.99 (0.92–1.06)	0.747

Data are presented as odds ratios (ORs) and 95% CIs. Each factor (sex [men versus women]; age [<55 years vs ≥55 years]; obstructive sleep apnea [OSA]; apnea‐hypopnea index [<15 events per hour versus ≥15 events per hour]; excessive daytime sleepiness [EDS; <11 versus ≥11]), and each continuous objective sleep parameter (slow wave sleep [SWS]; delta in nonrapid eye movement [NREM]; sigma in NREM; spindle density; and spindle aplitude) were succesivelly included together with an interaction term into the statistical model 2 adjusted for age, sex, body mass index, alcohol consumption, systolic blood pressure, diabetes, dyslipidemia, obstructive sleep apnea, sleep efficiency and duration of follow‐up.

## Discussion

This study investigated the associations between sleep macrostructure and microstructure and incident hypertension in a middle‐ to older population–based sample over a 5.2‐year follow‐up. Our results showed that sleep microstructural but not sleep macrostructural parameters were associated with incident hypertension. In particular, we found significant and robust associations between incident hypertension and low delta and low sigma power after multiple adjustments. Participants with values in the lowest quartile of delta and sigma had an ≈1.7‐fold increased incidence of hypertension compared with those with values in the highest quartile. Furthermore, we found that spindle density and amplitude were inversely associated with incident hypertension.

### Comparison With Previous Studies

Contrary to 2 previous community‐based studies that focused on sleep macrostructure,^14^, ^15^ we did not find an association between SWS and incident hypertension. The reason for this discrepancy remains uncertain but may be attributable to the lack of consistency of the classification method for human sleep stage scoring. Even if standard AASM rules are strictly followed,^32^ conventional sleep stage scoring is based on visual assessment of EEG, electrooculogram, and electromyogram data, based on arbitrary 30‐second epochs of sleep, which show considerable intrascorer and interscorer variability, particularly for N1 and SWS.^37^, ^38^, ^39^ Moreover, sleep staging is generally less reliable if sleep is fragmented, which commonly occurs in patients with sleep‐disordered breathing.^40^ Furthermore, conventional sleep stage scoring does not allow analysis of more subtle and potentially more clinically useful EEG features, such as the density, duration, and amplitude of spindles in NREM sleep.

Conversely, quantitative EEG provides a more objective and reliable alternative that is not prone to human error/variation to evaluate sleep structure using a continuous scale of sleep depth by classifying EEG according to its frequency content using automated and validated fast Fourier transformation–based algorithms. Interestingly, we found that higher sigma and delta power, which characterize important features of deep NREM sleep, were associated with reduced incidence of hypertension, independently of classical clinical covariates and obstructive sleep apnea, sleep efficiency, and systolic BP at baseline.

Our results confirm those of SWAN (Study of Women’s Health Across the Nation), a sleep study which showed that women with lower NREM delta power had a greater increase in diastolic BP during follow‐up and tended to be at increased risk for incident hypertension, independent of obstructive sleep apnea.^21^ However, to our knowledge, no previous studies have investigated other EEG power frequency bands. Thus, the present study is the first to demonstrate that not only slow wave activity (ie, power in the delta frequency band) but also faster brain activity (ie, power in the sigma frequency band reflecting spindles activity) in NREM sleep may protect against the development of hypertension. Furthermore, our findings extend the current literature by showing that some specific spindle characteristics, namely spindle density and amplitude, were also associated with a lower risk of incident hypertension after adjustment for multiple confounding factors.

### Potential Pathophysiological Mechanisms

The observed relationship between reduced slow wave activity (delta waves) and increased hypertension could reflect an underlying causal relationship with altered autonomic nervous system activity. Indeed, delta waves predominate during SWS, and studies investigating cardiac hemodynamics during sleep in healthy participants have shown decreased sympathetic activity and a concomitant increase in parasympathetic activity and baroreflex sensitivity with deeper sleep, which decreases BP and heart rate.^41^ As a result, nondipping BP associated with altered SWS could contribute to persistent elevation of daytime BP and further hypertension over the long term through mechanisms such as silent vascular damage, oxidative stress, and inflammation.^11^, ^42^

Sleep spindles are an EEG hallmark of N2 and are reflected in the sigma power frequency band.^43^ Although their functions are not clearly understood, observational and experimental evidence emphasize an important role of sleep spindles in memory consolidation and learning.^44^, ^45^ Higher sleep spindle density correlates with longer N2 sleep duration and greater resilience to sleep disruption from external perturbations.^43^, ^45^ At ages >40 years, studies even suggest that sleep spindles may become more relevant determinants of sleep quality than slow wave activity, which gradually decrease with age.^46^ Of interest, animal studies have shown that mice tend to wake up from sleep during spindle‐depleted periods, which may indicate a sleep “fragility period” where awakening is more likely.^47^ Conversely, spindle‐enriched periods, also called continuity periods, appear to be protective against arousals.^47^ Furthermore, autonomic control correlates with these fragility and continuity periods, and continuity periods with high spindle density are accompanied by lower heart rate and higher parasympathetic activity.^47^, ^48^ These observations may help to explain the observed protective effect of high sigma activity and spindle density in the development of hypertension. Last, although no longer consistent after multiple adjustments, the higher arousal index and proportion of N1 in bivariate analysis suggest that patiens who developed hypertension may have more fragmented sleep.

### Strengths and Limitations

The main strengths of this study are the large number of participants included and the unselected population‐based sample including both men and women, unlike SWAN.^21^ Compared with earlier studies that investigated the relationship between sleep structure and hypertension, we performed a more in‐depth analysis, assessing not only sleep macrostructure but also more comprehensive and objective sleep microstructural parameters using PSD EEG and novel spindle metrics. Moreover, comprehensive adjustment for covariates were assessed within the CoLaus study allowing for identification of robust independent associations between sleep metrics and incident hypertension.

Nevertheless, this study has some limitations. First, 24‐hour ambulatory BP monitoring data and nocturnal BP measurements would have been desirable. However, these measures are challenging in population cohorts and contribute to arousals and awakenings that disturb sleep quality likely to confound EEG metrics. Second, a familiarization night was not performed and the polysomnography equipment may have altered participants’ sleep quality even though polysomnography remains the gold standard to assess sleep. However, these effects would be expected to influence all participants relatively similarly. Furthermore, in‐home polysomnography, which allows participants to sleep in their usual environment, is likely to be less disturbing than sleep laboratory polysomnography. Third, one intrinsic limitation of the use of cortical EEG is related to the limited spatial resolution of this technique, which allows the measurements of signals close to the cortical surface. Although the central EEG derivations are the most common area analyzed, further studies are needed to determine whether our results are consistent across other cortical areas.^49^ Fourth, our study included almost exclusively White patients, which prevents the generalizability of our results to other ethnicities. Fifth, we acknowledge that baseline BP was higher in patients who developed hypertension during the follow‐up period, but this difference was addressed in the statistical analysis by adding baseline systolic BP as a covariate. Last, given the large sample size, it is likely that some differences, albeit statistically significant, are not clinically relevant. Nevertheless, the quartile analysis showed both statistically and clinically significant increased risk (from +69% to +124%) among participants with low delta power, sigma power, spindle density, and amplitude. As those sleep microstructure parameters have seldom been studied, it would be important that our results are replicated in other cohorts.

### Perspectives

Our findings indicate that sleep microstructural features are associated with incident hypertension. Slow wave activity and sleep spindles, 2 hallmarks of sleep continuity and objective sleep quality, were inversely associated with incident hypertension and were robust to multiple adjustments for potential confounders. These findings support the concept of a protective role of sleep continuity in the development of hypertension. However, additional studies are needed to confirm these results in other samples and to investigate potential underlying physiological mechanisms.

## Sources of Funding

The CoLaus study was supported by research grants from GlaxoSmithKline, the Faculty of Biology and Medicine of the University of Lausanne, and the Swiss National Science Foundation (grants 33CSCO‐122661, 33CS30‐139468, 33CS30‐148401, and 32473B‐182210). The HypnoLaus study received additional support from the Ligue Pulmonaire Vaudoise and the Leenaards Foundation. D.J.E. is supported by a National Health and Medical Research Council of Australia (NHMRC) Senior Research Fellowship (1116942) and an investigator grant (1196261).

## Disclosures

Outside the submitted work, D.J.E. has a Collaborative Research Centre grant, a consortium grant between the Australian government, Academia and Industry (Industry partner: Oventus Medical), and has research grants and serves as a consultant for Bayer, Takeda, and Invicta Medical. He also serves on the scientific advisory board for Apnimed. The remaining authors have no disclosures to report.

## Supporting information

Figure S1

## References

[1] Arima H , Barzi F , Chalmers J . Mortality patterns in hypertension. J Hypertens. 2011;29:S3–S7. doi: 10.1097/01.hjh.0000410246.59221.b1 22157565

[2] Hornyak M , Cejnar M , Elam M , Matousek M , Wallin BG . Sympathetic muscle nerve activity during sleep in man. Brain. 1991;114:1281–1295. doi: 10.1093/brain/114.3.1281 2065250

[3] Somers VK , Dyken ME , Mark AL , Abboud FM . Sympathetic‐nerve activity during sleep in normal subjects. N Engl J Med. 1993;328:303–307. doi: 10.1056/NEJM199302043280502 8419815

[4] Somers VK , Dyken ME , Clary MP , Abboud FM . Sympathetic neural mechanisms in obstructive sleep apnea. J Clin Invest. 1995;96:1897–1904. doi: 10.1172/JCI118235 7560081 PMC185826

[5] Ohkubo T , Hozawa A , Nagai K , Kikuya M , Tsuji I , Ito S , Satoh H , Hisamichi S , Imai Y . Prediction of stroke by ambulatory blood pressure monitoring versus screening blood pressure measurements in a general population: the Ohasama study. J Hypertens. 2000;18:847–854. doi: 10.1097/00004872-200018070-00005 10930181

[6] Dolan E , Stanton AV , Thom S , Caulfield M , Atkins N , McInnes G , Collier D , Dicker P , O'Brien E , Investigators A . Ambulatory blood pressure monitoring predicts cardiovascular events in treated hypertensive patients–an Anglo‐Scandinavian cardiac outcomes trial substudy. J Hypertens. 2009;27:876–885. doi: 10.1097/HJH.0b013e328322cd62 19516185

[7] Ben‐Dov IZ , Kark JD , Ben‐Ishay D , Mekler J , Ben‐Arie L , Bursztyn M . Predictors of all‐cause mortality in clinical ambulatory monitoring: unique aspects of blood pressure during sleep. Hypertension. 2007;49:1235–1241. doi: 10.1161/HYPERTENSIONAHA.107.087262 17389258

[8] Thurston RC , Chang Y , von Kanel R , Barinas‐Mitchell E , Jennings JR , Hall MH , Santoro N , Buysse DJ , Matthews KA . Sleep characteristics and carotid atherosclerosis among midlife women. Sleep. 2017;40. doi: 10.1093/sleep/zsw052 PMC6084762 28364498

[9] King CR , Knutson KL , Rathouz PJ , Sidney S , Liu K , Lauderdale DS . Short sleep duration and incident coronary artery calcification. JAMA. 2008;300:2859–2866. doi: 10.1001/jama.2008.867 19109114 PMC2661105

[10] Cappuccio FP , Cooper D , D'Elia L , Strazzullo P , Miller MA . Sleep duration predicts cardiovascular outcomes: a systematic review and meta‐analysis of prospective studies. Eur Heart J. 2011;32:1484–1492. doi: 10.1093/eurheartj/ehr007 21300732

[11] Javaheri S , Redline S . Sleep, slow‐wave sleep, and blood pressure. Curr Hypertens Rep. 2012;14:442–448. doi: 10.1007/s11906-012-0289-0 22846982

[12] Vivodtzev I , Tamisier R , Baguet JP , Borel JC , Levy P , Pepin JL . Arterial stiffness in COPD. Chest. 2014;145:861–875. doi: 10.1378/chest.13-1809 24687708

[13] Sayk F , Teckentrup C , Becker C , Heutling D , Wellhoner P , Lehnert H , Dodt C . Effects of selective slow‐wave sleep deprivation on nocturnal blood pressure dipping and daytime blood pressure regulation. Am J Physiol Regul Integr Comp Physiol. 2010;298:R191–R197. doi: 10.1152/ajpregu.00368.2009 19907004

[14] Fung MM , Peters K , Redline S , Ziegler MG , Ancoli‐Israel S , Barrett‐Connor E , Stone KL . Osteoporotic Fractures in Men Research G . Decreased slow wave sleep increases risk of developing hypertension in elderly men. Hypertension. 2011;58:596–603. doi: 10.1161/HYPERTENSIONAHA.111.174409 21876072 PMC3176739

[15] Javaheri S , Zhao YY , Punjabi NM , Quan SF , Gottlieb DJ , Redline S . Slow‐wave sleep is associated with incident hypertension: the sleep heart health study. Sleep. 2018:41. doi: 10.1093/sleep/zsx179 PMC5806562 29087522

[16] Schulz H . Rethinking sleep analysis. J Clin Sleep Med. 2008;4:99–103. doi: 10.5664/jcsm.27124 18468306 PMC2335403

[17] D'Rozario AL , Cross NE , Vakulin A , Bartlett DJ , Wong KKH , Wang D , Grunstein RR . Quantitative electroencephalogram measures in adult obstructive sleep apnea ‐ potential biomarkers of neurobehavioural functioning. Sleep Med Rev. 2017;36:29–42. doi: 10.1016/j.smrv.2016.10.003 28385478

[18] Appleton SL , Vakulin A , D'Rozario A , Vincent AD , Teare A , Martin SA , Wittert GA , McEvoy RD , Catcheside PG , Adams RJ . Quantitative electroencephalography measures in rapid eye movement and nonrapid eye movement sleep are associated with apnea‐hypopnea index and nocturnal hypoxemia in men. Sleep. 2019;42. doi: 10.1093/sleep/zsz092 31004167

[19] Parker JL , Melaku YA , D’Rozario AL , Wittert GA , Martin SA , Catcheside PG , Lechat B , Teare AJ , Adams RJ , Appleton SL , et al. The association between obstructive sleep apnea and sleep spindles in middle‐aged and older men: a community‐based cohort study. Sleep. 2022;45:zsab282. doi: 10.1093/sleep/zsab282 34850237

[20] Lechat B , Hansen KL , Melaku YA , Vakulin A , Micic G , Adams RJ , Appleton S , Eckert DJ , Catcheside P , Zajamsek B . A novel eeg derived measure of disrupted delta wave activity during sleep predicts all‐cause mortality risk. Ann Am Thorac Soc. 2022;19:649–658. doi: 10.1513/AnnalsATS.202103-315OC 34672877

[21] Matthews KA , Chang Y , Kravitz HM , Bromberger JT , Owens JF , Buysse DJ , Hall MH . Sleep and risk for high blood pressure and hypertension in midlife women: the SWAN (study of women's health across the nation) sleep study. Sleep Med. 2014;15:203–208. doi: 10.1016/j.sleep.2013.11.002 24360982 PMC3946296

[22] Gorgoni M , Lauri G , Truglia I , Cordone S , Sarasso S , Scarpelli S , Mangiaruga A , D’Atri A , Tempesta D , Ferrara M , et al. Parietal fast sleep spindle density decrease in Alzheimer's disease and amnesic mild cognitive impairment. Neural Plast. 2016;2016:8376108. doi: 10.1155/2016/8376108 27066274 PMC4811201

[23] Guadagni V , Byles H , Tyndall AV , Parboosingh J , Longman RS , Hogan DB , Hanly PJ , Younes M , Poulin MJ . Association of sleep spindle characteristics with executive functioning in healthy sedentary middle‐aged and older adults. J Sleep Res. 2021;30:e13037. doi: 10.1111/jsr.13037 32281182

[24] Firmann M , Mayor V , Vidal PM , Bochud M , Pécoud A , Hayoz D , Paccaud F , Preisig M , Song KS , Yuan X , et al. The CoLaus study: a population‐based study to investigate the epidemiology and genetic determinants of cardiovascular risk factors and metabolic syndrome. BMC Cardiovasc Disord. 2008;8:6. doi: 10.1186/1471-2261-8-6 18366642 PMC2311269

[25] Preisig M , Waeber G , Vollenweider P , Bovet P , Rothen S , Vandeleur C , Guex P , Middleton L , Waterworth D , Mooser V , et al. The PsyCoLaus study: methodology and characteristics of the sample of a population‐based survey on psychiatric disorders and their association with genetic and cardiovascular risk factors. BMC Psychiatry. 2009;9:9. doi: 10.1186/1471-244X-9-9 19292899 PMC2667506

[26] Heinzer R , Vat S , Marques‐Vidal P , Marti‐Soler H , Andries D , Tobback N , Mooser V , Preisig M , Malhotra A , Waeber G , et al. Prevalence of sleep‐disordered breathing in the general population: the Hypnolaus study. Lancet Respir Med. 2015;3:310–318. doi: 10.1016/S2213-2600(15)00043-0 25682233 PMC4404207

[27] Whelton PK , Carey RM , Aronow WS , Casey DE , Collins KJ , Dennison Himmelfarb C , DePalma SM , Gidding S , Jamerson KA , Jones DW , et al. 2017 ACC/AHA/AAPA/ABC/ACPM/AGS/APHA/ASH/ASPC/NMA/PCNA guideline for the prevention, detection, evaluation, and management of high blood pressure in adults: a report of the American College of Cardiology/American Heart Association task force on clinical practice guidelines. Hypertension. 2018;71:e13–e115. doi: 10.1161/HYP.0000000000000065 29133356

[28] American Diabetes Association . Diagnosis and classification of diabetes mellitus. Diabetes Care. 2014;37:S81–S90. doi: 10.2337/dc14-S081 24357215

[29] Allen RP , Picchietti D , Hening WA , Trenkwalder C , Walters AS , Montplaisi J . Restless Legs Syndrome Diagnosis and Epidemiology workshop at the National Institutes of Health; International Restless Legs Syndrome Study Group . Restless legs syndrome: diagnostic criteria, special considerations, and epidemiology. A report from the restless legs syndrome diagnosis and epidemiology workshop at the national institutes of health. Sleep Med. 2003;4:101–119. doi: 10.1016/s1389-9457(03)00010-8 14592341

[30] Buysse DJ , Reynolds CF III , Monk TH , Berman SR , Kupfer DJ . The Pittsburgh sleep quality index: a new instrument for psychiatric practice and research. Psychiatry Res. 1989;28:193–213. doi: 10.1016/0165-1781(89)90047-4 2748771

[31] Johns MW . A new method for measuring daytime sleepiness: the Epworth sleepiness scale. Sleep. 1991;14:540–545. doi: 10.1093/sleep/14.6.540 1798888

[32] Iber C , Ancoli‐Israel S , Chesson AL Jr , Quan SF . for the American Academy of Sleep Medicine . The AASM Manual for the Scoring of Sleep and Associated Events: Rules, Terminology and Technical Specifications. Westchester, IL: American Academy of Sleep Medicine; 2007.

[33] Berry RB , Budhiraja R , Gottlieb DJ , Gozal D , Iber C , Kapur VK , Marcus CL , Mehra R , Parthasarathy S , Quan SF , et al. Rules for scoring respiratory events in sleep: Update of the 2007 AASM manual for the scoring of sleep and associated events . Deliberations of the sleep apnea definitions task force of the American academy of sleep medicine. J Clin Sleep Med. 2012;8:597–619. doi: 10.5664/jcsm.2172 23066376 PMC3459210

[34] Lecci S , Cataldi J , Betta M , Bernardi G , Heinzer R , Siclari F . Electroencephalographic changes associated with subjective under‐ and overestimation of sleep duration. Sleep. 2020;43. doi: 10.1093/sleep/zsaa094 32409833

[35] Goldschmied JR , Lacourse K , Maislin G , Delfrate J , Gehrman P , Pack FM , Staley B , Pack AI , Younes M , Kuna ST , et al. Spindles are highly heritable as identified by different spindle detectors. Sleep. 2021;44. doi: 10.1093/sleep/zsaa230 PMC8033448 33165618

[36] Carrier J , Semba K , Deurveilher S , Drogos L , Cyr‐Cronier J , Lord C , Sekerovick Z . Sex differences in age‐related changes in the sleep‐wake cycle. Front Neuroendocrinol. 2017;47:66–85. doi: 10.1016/j.yfrne.2017.07.004 28757114

[37] Norman RG , Pal I , Stewart C , Walsleben JA , Rapoport DM . Interobserver agreement among sleep scorers from different centers in a large dataset. Sleep. 2000;23:901–908. doi: 10.1093/sleep/23.7.1e 11083599

[38] Magalang UJ , Chen NH , Cistulli PA , Fedson AC , Gíslason T , Hillman D , Penzel T , Tamisier R , Tufik S , Phillips G , et al. Agreement in the scoring of respiratory events and sleep among international sleep centers. Sleep. 2013;36:591–596. doi: 10.5665/sleep.2552 23565005 PMC3612261

[39] Younes M , Kuna ST , Pack AI , Walsh JK , Kushida CA , Staley B , Pien GW . Reliability of the American academy of sleep medicine rules for assessing sleep depth in clinical practice. J Clin Sleep Med. 2018;14:205–213. doi: 10.5664/jcsm.6934 29351821 PMC5786839

[40] Danker‐Hopfe H , Kunz D , Gruber G , Klösch G , Lorenzo JL , Himanen SL , Kemp B , Penzel T , Röschke J , Dorn H , et al. Interrater reliability between scorers from eight European sleep laboratories in subjects with different sleep disorders. J Sleep Res. 2004;13:63–69. doi: 10.1046/j.1365-2869.2003.00375.x 14996037

[41] Trinder J , Kleiman J , Carrington M , Smith S , Breen S , Tan N , Kim Y . Autonomic activity during human sleep as a function of time and sleep stage. J Sleep Res. 2001;10:253–264. doi: 10.1046/j.1365-2869.2001.00263.x 11903855

[42] Pierdomenico SD , Costantini F , Bucci A , De Cesare D , Bucciarelli T , Cuccurullo F , Mezzetti A . Blunted nocturnal fall in blood pressure and oxidative stress in men and women with essential hypertension. Am J Hypertens. 1999;12:356–363. doi: 10.1016/S0895-7061(98)00273-8 10232495

[43] Purcell SM , Manoach DS , Demanuele C , Cade BE , Mariani S , Cox R , Panagiotaropoulou G , Saxena R , Pan JQ , Smoller JW , et al. Characterizing sleep spindles in 11,630 individuals from the national sleep research resource. Nat Commun. 2017;8:15930. doi: 10.1038/ncomms15930 28649997 PMC5490197

[44] De Gennaro L , Ferrara M . Sleep spindles: an overview. Sleep Med Rev. 2003;7:423–440. doi: 10.1053/smrv.2002.0252 14573378

[45] Fernandez LM , Luthi A . Sleep spindles: mechanisms and functions. Physiol Rev. 2020;100:805–868. doi: 10.1152/physrev.00042.2018 31804897

[46] Landolt HP , Dijk DJ , Achermann P , Borbely AA . Effect of age on the sleep eeg: slow‐wave activity and spindle frequency activity in young and middle‐aged men. Brain Res. 1996;738:205–212. doi: 10.1016/S0006-8993(96)00770-6 8955514

[47] Yuzgec O , Prsa M , Zimmermann R , Huber D . Pupil size coupling to cortical states protects the stability of deep sleep via parasympathetic modulation. Curr Biol. 2018;28:392 – 400.e393. doi: 10.1016/j.cub.2017.12.049 29358069 PMC5807087

[48] Lecci S , Fernandez LM , Weber FD , Cardis R , Chatton JY , Born J , Luthi A . Coordinated infraslow neural and cardiac oscillations mark fragility and offline periods in mammalian sleep. Sci Adv. 2017;3:e1602026. doi: 10.1126/sciadv.1602026 28246641 PMC5298853

[49] Ruehland WR , O'Donoghue FJ , Pierce RJ , Thornton AT , Singh P , Copland JM , Stevens B , Rochford PD . The 2007 AASM recommendations for eeg electrode placement in polysomnography: impact on sleep and cortical arousal scoring. Sleep. 2011;34:73–81. doi: 10.1093/sleep/34.1.73 21203376 PMC3001799

