# Supplementary material for: Association Between Sleep Microstructure and Incident Hypertension in a Population‐Based Sample: The HypnoLaus Study
Source: J Am Heart Assoc. 2022 Jul 5;11(14):e025828. doi: 10.1161/JAHA.121.025828 (PMC9707830; doi:10.1161/JAHA.121.025828)
Supplement: Supplementary file 1 — Figure S1 [file JAH3-11-e025828-s001.pdf]

SUPPLEMENTAL MATERIAL

Figure S1. Restricted cubic spline regression for the association between spindle density and incident hypertension.

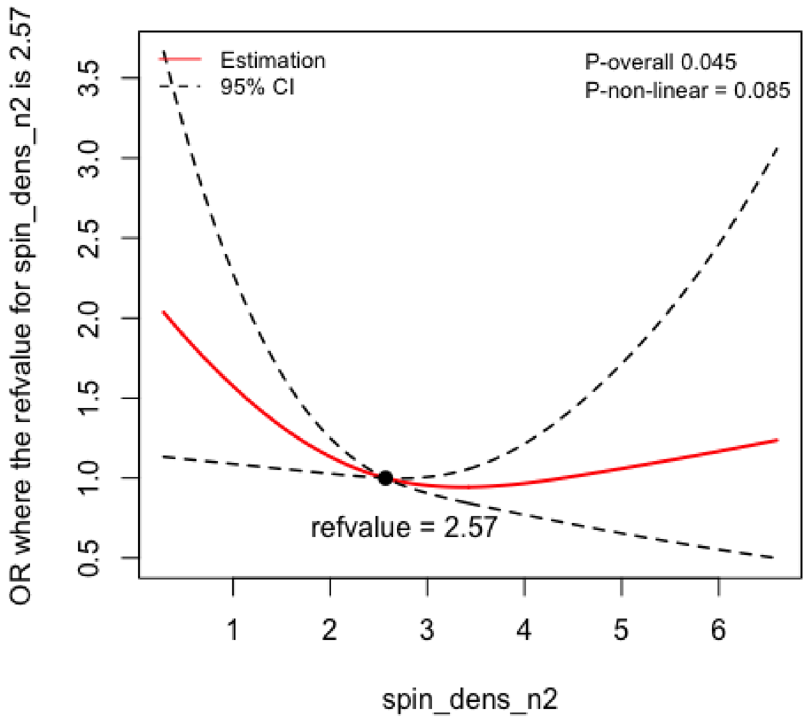

The red solid line represents the OR and the black dashed lines represent the 95% confidence intervals. Knots were placed at the 05th, 50th and 95th percentiles of spindle density. The reference value was 2.57. The model was adjusted for baseline age, body mass index, sex, alcohol consumption, systolic blood pressure, diabetes, dyslipidemia, obstructive sleep apnea, and sleep efficiency (Model 2).
